# Association of public health insurance with cancer-specific mortality risk among patients with nasopharyngeal carcinoma: a prospective cohort study in China

**DOI:** 10.3389/fpubh.2023.1020828

**Published:** 2023-06-02

**Authors:** Dan Li, Hai-Ke Lei, Xiao-Lei Shu, Xin Zhang, Hong-Lei Tu, Feng Wang, Yu-Wei Wang, Ying Wang, Jiang-Dong Sui

**Affiliations:** ^1^School of Medicine, Chongqing University, Chongqing, China; ^2^Chongqing Cancer Multi-omics Big Data Application Engineering Research Center, Chongqing University Cancer Hospital, Chongqing, China; ^3^Radiation Oncology Center, Chongqing University Cancer Hospital, Chongqing, China

**Keywords:** health insurance, nasopharyngeal carcinoma, NPC-specific mortality, self-paying rate, NPC prognosis

## Abstract

**Objective:**

Health insurance programs are effective in preventing financial hardship in patients with cancer. However, not much is known about how health insurance policies, especially in Southwest China with a high incidence of nasopharyngeal carcinoma (NPC), influence patients’ prognosis. Here, we investigated the association of NPC-specific mortality with health insurance types and self-paying rate, and the joint effect of insurance types and self-paying rate.

**Materials and methods:**

This prospective cohort study was conducted at a regional medical center for cancer in Southwest China and included 1,635 patients with pathologically confirmed NPC from 2017 to 2019. All patients were followed up until May 31, 2022. We determine the cumulative hazard ratio of all-cause and NPC-specific mortality in the groups of various insurance kinds and the self-paying rate using Cox proportional hazard.

**Results:**

During a median follow-up period of 3.7 years, 249 deaths were recorded, of which 195 deaths were due to NPC. Higher self-paying rate were associated with a 46.6% reduced risk of NPC-specific mortality compared to patients with insufficient self-paying rate (HR: 0.534, 95% CI: 0.339–0.839, *p* = 0.007). For patients covered by Urban and Rural Residents Basic Medical Insurance (URRMBI), and for patients covered by Urban Employee Basic Medical Insurance, each 10% increase in the self-paying rate reduced the probability of NPC-specific death by 28.3 and 25%, respectively (UEBMI).

**Conclusion:**

Results of this study showed that, despite China’s medical security administration improved health insurance coverage, NPC patients need to afford the high out-of-pocket medical costs in order to prolong their survival time.

## Introduction

Nasopharyngeal carcinoma (NPC) has a notable ethnic and geographic distribution, with a predominance in southern China and southeast Asia (1). Although NPC is fairly uncommon in several jurisdictions, it remains a significant public health problem in South China, which accounted for more than 40% of the approximate 130,000 patients worldwide in 2020 (2, 3). Radiotherapy or chemoradiotherapy is considered the primary radical treatment for non-metastatic NPC and the 3-year failure-free survival rate for non-metastatic patients exceeds 80% (4–6). Cancer survival now means living with a chronic and complex condition. Thus, the long-term management and care for cancer survivors bring severe financial stress to individuals and their families, particularly in the great majority of less developed countries where the public healthcare system is inadequate to lessen the burden on citizens (7, 8).

The presence of medical insurance system is considered a means of reducing the financial burden of cancer prevention and treatment, which seems essential for a country to achieve universal healthcare coverage and health equity. The majority of studies on the correlation between medical insurance and cancers have focused on geographical variations in health insurance coverage for cancer patients (9, 10), differences in the financial burden of cancer patients by type of health insurance (11–15), and the coverage of new types of anti-cancer treatments by health insurance policies (16–19). Fewer shreds of evidence have shown the influence of current medical insurance policies on cancer prognosis. So far, only studies on liver (20), lung (21), and breast cancers (22, 23) have suggested that underinsured patients have an increased risk of death and patients require higher out-of-pocket costs to reduce this risk. However, it is unknown whether the medical insurance policies, especially in high incidence areas of NPC, where the patients may afford higher self-paying rate, influence cancer prognosis. Therefore, we sought to investigate the association between health insurance types and self-paying rate and the incidence of NPC-specific death, as well as the joint effect of insurance types and self-paying rate, using a prospective cohort of patients diagnosed with NPC from 2017 to 2019.

## Materials and methods

### Patients selection

The medical database of Chongqing University Cancer Hospital covers almost all patients with nasopharyngeal carcinoma diagnosed in Chongqing since 2017, which is the foundation of this prospective cohort study. We enrolled 1,635 patients between May 31, 2017 and May 31, 2019. Clinical records were independently reviewed through the hospital’s electronic medical record database to prospectively gather data on demographic and clinical traits, lab tests, and treatments. In addition, patients with the following characteristics were excluded: (1) Non-primary NPC, (2) Pathologically confirmed non-squamous cell carcinoma, (3) Most of the treatment was not performed at our institution, (4) Missing data information collected, (5) Minors (17 years old and younger), and (6) No follow-up records.

### Health insurance

China’s basic medical insurance is a national, provincial, municipal, and county-level system. The Urban Employee Basic Medical Insurance Scheme (UEBMI), which covers only unit employees, was implemented in 1998. The New Rural Cooperative Medical Scheme (NRCMS), which covers rural residents, was implemented in 2003. The Urban Residents Basic Medical Insurance Scheme (URBMI), which covers urban non-workers (students, children and youth, non-working urban residents, etc.), was implemented in 2007. URBMI and NRCMS merged in 2016 to create a basic medical insurance plan for urban and rural residents (URRBMI). Both types of medical insurance cover the same services, but their reimbursement ratios differ. As of 2019, the UEBMI reimbursement ratio in Chongqing is 83% and the URRBMI ratio is 75%. This difference is due to different insurance contributions. Chongqing’s 2020 URRBMI contribution rate is 220 or 550 RMB/person-years, but UEBMI requires a minimum health insurance contribution of 4,189 RMB/person-years, 20% of which is paid by the individual and the remainder is paid by the employer. Due to different contribution methods, reimbursement comes from different places. Individual contributions and government subsidies fund URRBMI, while UEBMI has individual accounts (individual contribution plus a small portion of unit contribution) and coordinated funds (the majority of the unit portion of the contribution plus government subsidies, funding, etc.). Geography (inside and outside the city), contribution status (contribution interruption, contribution years), and hospital level also affect reimbursement rate (Level I, II, III). The municipal government coordinates the regional implementation of health insurance reimbursement’s complex calculation. During treatment, patients must pay for medications, treatment items, and medical services that are not covered by their basic medical insurance, and the self-paying rate is the proportion of this cost to the patient’s total treatment cost.

The Chongqing University Cancer Hospital’s information system (HIS) provides data on the types of medical insurance and hospitalization expenses. Due to the time window of medical insurance policy implementation, URRBMI in this study refers to both URMBI and URRBMI medical insurance types, which differ in terms of administration and reimbursement rate insignificantly. During the statistical analysis, there were only 44 NRCMS, so they were excluded from the study; additionally, 393 patients were publicly funded, self-funded, covered by commercial insurance, or had blank registration information, so they were excluded from the study; 11 patients with unclear costs were also excluded. For further analysis of health insurance efficacy, by referring to the literature of similar studies (21, 22) in conjunction with our study, we calculated median values for health self-paying rate (56.8% for URRBMI and 46.8% for UEBMI), and we divided the self-paying rate into a low (0–50%) and a high (> 50%) group for inclusion in the analysis.

### Outcome and follow-up

The primary outcome in this study was NPC-specific death, and the secondary outcome was all-cause death. Patients were followed up from the day they were diagnosed with NPC until the event (death) occurred or the follow-up cut-off time passed (May 31, 2022). The follow-up data were obtained from the records of patients’ voluntary follow-up visits to our hospital and telephone follow-ups received from our hospital at 3–6 months intervals by patients or their immediate family members. Multiple relatives were sought in cases of death, and medical records were thoroughly examined to adequately define the type of death and reduce research error.

### Statistical analysis

First, we ran descriptive statistics on patient demographic and clinical baseline data, reporting count data as frequencies and percentages. Demographic and clinical baseline characteristics were counted for each insurance type and self-paying rate grouping, and differences were examined using *chi-square* tests. After controlling for demographic and clinical factors, we used logistic regression to investigate the relationship between health insurance type, out-of-pocket costs, and several NPC treatment modalities (radiotherapy, chemotherapy, and targeted therapy). Subsequently, we used Cox regression models to compare the hazard ratios (HRs) and 95% confidence intervals for cancer-specific and all-cause mortality for patients with URRMBI and UEMBI, as well as for NPC patients with less than 50% and greater than or equal to 50% self-paying rate. Finally, the impacts of medical insurance type and self-paying rate on mortality risk were examined for joint effects, as well as the link between each 10% rise in deductible and the probability of death from any cause as well as from NPC-specific causes was examined.

We built three models in analysis to reduce the possibility of confounding bias from other factors. We adjusted for demographic factors and tumor characteristics such as age, gender, ethnic group, marital status, occupation, tumor stage, and tumor histopathological classification in model A. Model B was based on model A, with additional adjustments for potential clinical indicators affecting the prognosis of NPC patients including such plasma EBV viral DNA load, serum albumin to globulin ratio(A/G) (24), lactate dehydrogenase to albumin ratio(LAR) (25), peripheral blood neutrophil count to lymphocyte count ratio(NLR) (26), and peripheral blood platelet count to lymphocyte count ratio(PLR) (25). Similar to model B, model C was based on it and controls for different treatment modalities, like radiotherapy, chemotherapy, and targeted therapy. Before including the data on clinical continuous variables in the analysis, we categorized them by reviewing the literature or using X-tile software to find truncation values.

Software R (version 4.0.2; R Foundation for Statistical Computing, Vienna, Austria) and SAS9.4 statistical software (version 9.4; SAS Institute Inc., Cary, North Carolina) were used to analyze all of the data. *p* < 0.05 indicates a statistically significant difference.

## Results

### Patients’ characteristics

Applying the inclusion and exclusion standards, there were 1,635 patients with NPC of which 446 (27.28%) were females and 1,189 (72.72%) were males in the final cohort. They were almost Han and only 221 were minorities, with a median age of 51 years old. Their tumors were more likely to be non-metastatic (91.31%), undifferentiated (40.37%), and to have the locally advanced stage. Among the included study subjects, 1,055 (64.5%) patients were insured by URBMI, and the remaining 580 (35.5%) patients were insured by UEBMI. The median self-paying rate in URRBMI was 56.8%, whereas it was 46.8% in UEBMI. When categorized at a 50% deductible, 667 patients were below 50%, while the remaining 968 patients were above or just 50%. Most of the younger patients were covered by UEBMI, and there were no differences in marital status and gender between the different insurance types. Clinical stage and EBV copy number were statistically significant in the self-paying rate group. Table 1 shows the full study results.

**Table 1:** NPC patient characteristics by insurance type and self-paying rate.

		By insurance type			By self-paying rate		
	ALL (*N* = 1,635) N (%)	URRBMI (*N* = 1,055) N (%)	UEBMI (*N* = 580) *N* (%)	*p*	≤50% (*N* = 667) *N* (%)	>50% (*N* = 968) *N* (%)	*p*
Age at diagnosis, years				<0.001			0.748
18–40	233 (14.25)	123 (11.66)	110 (18.97)		90 (13.49)	143 (14.77)	
41–50	503 (30.76)	345 (32.7)	158 (27.24)		205 (30.73)	298 (30.79)	
≥51	899 (54.98)	587 (55.64)	312 (53.79)		372 (55.77)	527 (54.44)	
Gender				0.285			0.826
Female	446 (27.28)	297 (28.15)	149 (25.69)		180 (26.99)	266 (27.48)	
Male	1,189 (72.72)	758 (71.85)	431 (74.31)		487 (73.01)	702 (72.52)	
Ethnic group				<0.001			<0.0001
Han	1,414 (86.48)	983 (93.18)	431 (74.31)		541 (81.11)	873 (90.19)	
Minority	221 (13.52)	72 (6.82)	149 (25.69)		126 (18.89)	95 (9.81)	
Marital status				0.618			0.429
Married	1,507 (92.17)	975 (92.42)	532 (91.72)		619 (92.8)	888 (91.74)	
Another status^a^	128 (7.83)	80 (7.58)	48 (8.28)		48 (7.2)	80 (8.26)	
Occupation				<0.001			<0.0001
Employees/workers	289 (17.68)	143 (13.55)	146 (25.17)		137 (20.54)	152 (15.7)	
Non-practitioners^b^	598 (36.57)	470 (44.55)	128 (22.07)		162 (24.29)	436 (45.04)	
Special Employees^c^	141 (8.62)	34 (3.22)	107 (18.45)		97 (14.54)	44 (4.55)	
Other professional	607 (37.13)	408 (38.67)	199 (34.31)		271 (40.63)	336 (34.71)	
Cancer stage^d^				<0.001			0.010
I	18 (1.1)	6 (0.57)	12 (2.07)		8 (1.2)	10 (1.03)	
II	244 (14.92)	133 (12.61)	111 (19.14)		116 (17.39)	128 (13.22)	
III	673 (41.16)	433 (41.04)	240 (41.38)		288 (43.18)	385 (39.77)	
IV	700 (42.81)	483 (45.78)	217 (37.41)		255 (38.23)	445 (45.97)	
Cancer metastasis status				0.945			0.583
Non-metastatic	1,493 (91.31)	963 (91.28)	530 (91.38)		606 (90.85)	887 (91.63)	
Metastatic	142 (8.69)	92 (8.72)	50 (8.62)		61 (9.15)	81 (8.37)	
Histological keratinization status^e^				0.475			0.88
Non-keratinized differentiated	953 (58.29)	626 (59.34)	327 (56.38)		392 (58.77)	561 (57.95)	
Non-keratinized undifferentiated	660 (40.37)	416 (39.43)	244 (42.07)		267 (40.03)	393 (40.6)	
Keratinized and other	22 (1.35)	13 (1.23)	9 (1.55)		8 (1.2)	14 (1.45)	
EBV-DNA, copy/mL				0.053			<0.001
<1,000	923 (56.45)	577 (62.51)	346 (37.49)		440 (65.97)	483 (49.9)	
≥1,000	712 (43.55)	478 (67.13)	234 (32.87)		227 (34.03)	485 (50.1)	
A/G				0.308			0.95
≤0.13	178 (10.89)	121 (67.98)	57 (32.02)		73 (10.94)	105 (10.85)	
>0.13	1,457 (89.11)	934 (64.1)	523 (35.9)		594 (89.06)	863 (89.15)	
LAR				0.537			0.501
≤6.15	1,438 (87.95)	924 (64.26)	514 (35.74)		591 (88.61)	847 (87.5)	
>6.15	197 (12.05)	131 (66.5)	66 (33.5)		76 (11.39)	121 (12.5)	
NLR				0.495			0.886
≤4.84	1,444 (88.32)	936 (64.82)	508 (35.18)		590 (88.46)	854 (88.22)	
>4.84	191 (11.68)	119 (62.3)	72 (37.7)		77 (11.54)	114 (11.78)	
PLR				0.149			0.23
≤206.33	1,290 (78.9)	821 (63.64)	469 (36.36)		536 (80.36)	754 (77.89)	
>206.33	345 (21.1)	234 (67.83)	111 (32.17)		131 (19.64)	214 (22.11)	

a Unmarried/divorced/widowed and/or other.

b Self-employed /Unemployed/ Freelance/Students /Farmers.

c Retired (retired) staff/civil servants/Professional and technical staff.

d Referring to the 8th edition of the UICC/AJCC staging system.

e Referring to the World Health Organization 2005 classification of head and neck tumors.

### Health insurance type, self-paying rate, and NPC treatment

We investigated the association of the groups of different health insurance types and self-paying rate, with treatment options for NPC, using the grouping of insurance type and self-paying rate as independent variables, treatment regimens as the outcome, and other adjustment variables in the model. As shown in Table 2, after adjusting gender, occupation, marital status, ethnicity, age at diagnosis, cancer stage, histopathological diagnosis and clinical laboratory indicators, patients insured by UEBMI are more likely to choose radiotherapy, chemotherapy, targeted therapy when compared with patients who were insured by URRBM. Moreover, choosing these treatment regimens (including radiotherapy, chemotherapy, and targeted therapy) could obviously lead to higher out-pocket costs for NPC patients.

**Table 2:** Associations between treatment kind and insurance type and self-paying rate, according to demographic and clinical traits.

		Model A^a^		Model B^b^		Model C^c^	
	Number of events (%)^d^	OR (95%CI)	*p*	OR (95%CI)	*p*	OR (95%CI)	*p*
Radiotherapy							
By insurance type							
URRBMI	595 (56.4)	1		1		1	
UEBMI	359 (61.9)	1.433 (1.136–1.807)	0.002	1.433 (1.135–1.810)	0.003	1.415 (1.115–1.795)	0.004
By self-paying rate							
≤50%	287 (43.03)	1		1		1	
>50%	667 (68.9)	2.898 (2.335–3.596)	<0.001	2.955 (2.377–3.673)	<0.001	2.843 (2.278–3.548)	<0.001
Chemotherapy							
By insurance type							
URRBMI	635 (60.19)	1		1		1	
UEBMI	378 (65.17)	1.373 (1.084–1.739)	0.009	1.413 (1.113–1.794)	0.004	1.408 (1.101–1.800)	0.006
By self-paying rate							
≤50%	311 (46.63)	1		1		1	
>50%	702 (72.52)	3.028 (2.431–3.773)	<0.001	3.026 (2.426–3.774)	<0.001	2.900 (2.312–3.638)	<0.001
Targeted therapy							
By insurance type							
URRBMI	261 (24.74)	1		1		1	
UEBMI	177 (30.52)	1.439 (1.123–1.844)	0.004	1.564 (1.215–2.014)	0.001	1.549 (1.198–2.002)	0.001
By self-paying rate							
≤50%	124 (18.59)	1		1		1	
>50%	314 (32.44)	2.088 (1.630–2.674)	<0.001	2.063 (1.606–2.650)	<0.001	1.915 (1.483–2.471)	<0.001

a The odds ratios were adjusted for demographic characteristics such as diagnostic age, gender, ethnic group, marital status, and occupation.

b The odds ratios were additionally adjusted for cancer characteristics, such as cancer stage, and cancer histopathological type.

c The odds ratios were additionally adjusted for potential clinical such as affecting the prognosis of NPC, including EBV-DNA load, A/G, LAR, NLR, and PLR.

d Number of events as a percentage of the number of classified patients.

### NPC-specific mortality and health insurance type and self-paying rate

**Figure 1:**
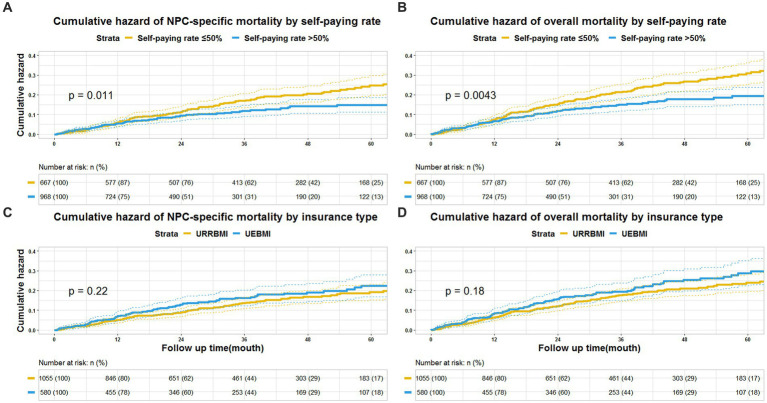
Cumulative hazard in patients with nasopharyngeal carcinoma. **(A)** Cumulative hazard of NPC-specific mortality by self-paying rate (self-paying rate). **(B)** Cumulative hazard of overall mortality self-paying rate (self-paying rate). **(C)** Cumulative hazard of NPC-specific mortality by insurance type. **(D)** Cumulative hazard of overall mortality by insurance type.

During a median follow-up period of 3.7 years, 249 deaths were recorded, of which 195 died of NPC. As shown in Figure 1, Patients with a lower self-paying rate had a greater cumulative hazard overall and NPC-specific mortality than patients with a higher self-paying rate. The overall and NPC-specific mortality rate were not different between patients receiving UEBMI and those with URRBMI. After adjusting for demographic and tumor characteristics, patients with higher out-of-pocket costs compared to those with insufficient out-of-pocket costs experienced a risk reduction of overall mortality of 56.7% (HR: 0.433, 95% CI: 0.322–0.583, *p* < 0.001) and a risk reduction of NPC-specific mortality of 61.4% (HR: 0.386, 95% CI: 0.253–0.588, *p* < 0.001) (Table 3). When additionally adjusting the clinical characteristics and NPC treatment regimens, the risk of overall mortality was reduced by 47.5% (HR: 0.525, 95% CI: 0.380–0.727, *p* < 0.001) for patients with higher self-paying rate in comparison to patients with insufficient self-paying rate, and the risk of NPC-specific mortality was reduced by 46.6% (HR: 0.534, 95% CI: 0.339–0.839, *p* = 0.007).

**Table 3:** Associations between insurance type and self-paying rate and NPC-specific or overall mortality risks.

	Number of patients	Number of events	Rate %	Model A^a^		Model B^b^		Model C^c^	
				HR (95%CI)	*p*	HR (95%CI)	*p*	HR (95%CI)	*p*
Overall mortality									
By insurance type									
URRBMI	1,055	151	5.04	1		1		1	
UEBMI	580	98	5.95	1.101 (0.799–1.517)	0.557	1.107 (0.792–1.547)	0.551	1.200 (0.846–1.703)	0.307
By self-paying rate									
≤50%	667	144	6.22	1		1		1	
>50%	968	105	4.93	0.433 (0.322–0.583)	<0.001	0.384 (0.282–0.525)	<0.001	0.525 (0.380–0.727)	<0.001
NPC-specific mortality									
By insurance type									
URRBMI	1,055	118	3.94	1		1		1	
UEBMI	580	77	4.68	1.294 (0.830–2.017)	0.255	1.277 (0.806–2.021)	0.298	1.370 (0.843–2.228)	0.204
By self-paying rate									
≤50%	667	113	4.88	1		1		1	
>50%	968	82	3.85	0.386 (0.253–0.588)	<0.001	0.349 (0.226–0.540)	<0.001	0.534 (0.339–0.839)	0.007

a The hazard ratios were adjusted for demographic factors and cancer characteristics, such as diagnostic age, gender, ethnic group, marital status, occupation, cancer stage, and cancer histopathological type.

b The hazard ratios were additionally adjusted for potential clinical indicators affecting the prognosis of NPC, such as EBV-DNA load, A/G, LAR, NLR, and PLR.

c The hazard ratios were additionally adjusted for different treatment modalities, such as radiotherapy, chemotherapy, and targeted therapy.

### The joint effect of insurance types and Self-paying rate

We demonstrated that each 10% increase in the self-paying rate was related to a 24.3% decreased overall risk of NPC-specific mortality (HR: 0.757, 95% CI: 0.647–0.887, *p* < 0.001), and a 28.3% decreased risk of NPC-specific mortality for patients receiving URRBMI (HR: 0.717, 95% CI: 0.611–0.842, *p* < 0.001), and a 25% decreased risk of NPC-specific mortality for patients receiving UEBMI (HR: 0.750, 95% CI: 0.626–0.899, *p* = 0.002). Similar results in overall mortality were discovered (Table 4).

**Table 4:** Association between each 10% increase in insurance self-paying rate and risk of specific or overall mortality from NPC.

	Number of patients	Number of events	Rate %	Model A^a^		Model B^b^		Model C^c^	
				HR (95%CI)	*p*	HR (95%CI)	*p*	HR (95%CI)	*p*
Overall mortality									
Any insurance type									
Per 10% increase	1,635	249	5.37	0.941 (0.850–1.042)	0.242	0.884 (0.796–0.982)	0.021	0.849 (0.762–0.947)	0.003
Within URRBMI									
Per 10% increase	1,055	151	5.04	0.932 (0.842–1.031)	0.17	0.902 (0.815–0.999)	0.047	0.863 (0.777–0.958)	0.006
Within UEBMI									
Per 10% increase	580	98	5.95	0.833 (0.740–0.938)	0.002	0.787 (0.695–0.891)	<0.001	0.738 (0.645–0.844)	<0.001
NPC-specific mortality									
Any insurance type									
Per 10% increase	1,635	195	4.20	0.827 (0.711–0.962)	0.014	0.778 (0.667–0.907)	0.001	0.757 (0.647–0.887)	<0.001
Within URRBMI									
Per 10% increase	1,055	118	3.94	0.789 (0.674–0.925)	0.003	0.765 (0.655–0.895)	0.001	0.717 (0.611–0.842)	<0.001
Within UEBMI									
Per 10% increase	580	77	4.68	0.817 (0.691–0.965)	0.018	0.776 (0.653–0.921)	0.004	0.750 (0.626–0.899)	0.002

a The hazard ratios were adjusted for demographic factors and cancer characteristics, such as diagnostic age, gender, ethnic group, marital status, occupation, cancer stage, and cancer histopathological type.

b The hazard ratios were additionally adjusted for potential clinical indicators affecting the prognosis of NPC, such as EBV-DNA load, A/G, LAR, NLR, and PLR.

c The hazard ratios were additionally adjusted for different treatment modalities, such as radiotherapy, chemotherapy, and targeted therapy.

## Discussion

To the extent that we are aware, this is the first study to comprehensively analyze the relationship between all-cause and NPC-specific death and insurance types or self-paying rate in NPC patients in China. In this large-scale cohort of 1,635 NPC patients treated at Chongqing University Cancer Hospital from 2017 to 2019, the sub-group analysis was conducted based on insurance types and self-paying rate. We found that the greater self-paying rate is associated with the lower incidence of NPC-specific death. Importantly, our results strongly suggest that the incidence of NPC-specific death was reduced by 25% per 10% rise in UEBMI’s self-paying rate, while the incidence of NPC-specific death was reduced by 28.3% per 10% rise in URRBMI’s self-paying rate. These associations are understood in part but not entirely by proven prognostic factors such as clinical features and NPC treatment regimens.

With the continuous development of precision radiotherapy technology and anti-cancer drugs, NPC patients have a great clinical curative effect and extended survival time, but meanwhile it also further increases the financial burden on individuals and their families (6). Since costly diagnostic screening (e.g., PET-CT), precise radiotherapy regimens (e.g., Helical Tomotherapy), and a part of anti-cancer targeted drugs and immune checkpoint inhibitors were not covered by medical insurance lists in China, NPC patients must afford the high out-of-pocket costs in order to prolong their survival time and improve their survival quality. In fact, our analysis shown that the association between high self-paying rate and NPC-specific mortality is somewhat attenuated by close to 15% after adjusting for treatment characteristics, suggesting a contribution from existing cancer treatments. Our data therefore provide a unique opportunity to uncover the underlying mechanisms of the relationship between health insurance and cancer prognosis, emphasizing the critical need to broaden the scope of reimbursement for diagnosis and treatment along with drugs in health insurance in order to dramatically enhance the prognosis of patients and minimize their financial hardship.

Patients with inadequate insurance would face a heavy economic burden and will not be able to pay for advanced therapy out-of-pocket (27). It is plausible that suboptimal medical services may lead to compromised prognosis after cancer diagnosis (28). Indeed, independent of clinical factors, our investigation showed that patients receiving URRBMI were more likely to have an advanced disease stage and that patients with low self-paying rate received less radiation, chemotherapy, and targeted treatment. For instance, Helical Tomotherapy was not covered by medical insurance lists during the study period, and the high out-of-pocket medical expenses may prevent financially vulnerable patients from such precise radiotherapy.

The increased prevalence of NPC-specific mortality in patients with insufficient out-of-pocket payments is not only due to differences in tumor features and treatment modalities. This is supported by our data that the increased risk of NPC-specific death remained robust in patients with low self-paying rate after exhaustive adjustment for clinical factors and treatment regimens. In addition, it is known that cancer patients face enormous psychological stress during cancer diagnosis and treatment (29). The high out-of-pocket costs, as a consequence of inadequate insurance, may aggravate patients’ emotional turmoil. It is no doubt that financial burden may impact patients’ prognosis *via* psychological disorders.

One significant feature of our study is that it is the first to investigate the relationship between NPC patients’ prognosis and medical expenses using a prospective cohort study design with comprehensive follow-up data. The abundance of data on the patient’s clinical and demographic traits helped us highlight how clearly health insurance policies influence NPC-specific mortality, which offers a fresh perspective for the development of our nation’s medical insurance. This study, however, has certain limitations. To begin with, because this cohort is based on a regional cancer center, the results can only provide a reference for generalization to the entire population. Second, our cohort only recorded the expenses incurred in the hospital for the treatment of NPC patients, but a small part of the costs incurred outside the hospital cannot be estimated. Third, we only discuss the association of NPC-specific mortality with insurance types and self-paying rate in the present study. Finally, the possible lack of patients who are most financially vulnerable may lead to information bias.

In conclusion, our findings suggest that patients with lower out-of-pocket costs face a higher risk of NPC-specific mortality in China, which may provide novel insights into the role of self-paying rate in cancer health disparities in China and likewise developing countries.

## Data availability statement

The raw data supporting the conclusions of this article will be made available by the authors, without undue reservation.

## Ethics statement

Ethical review and approval was not required for the study on human participants in accordance with the local legislation and institutional requirements. Written informed consent from the patients was not required to participate in this study in accordance with the national legislation and the institutional requirements.

## Author contributions

YW, J-DS, and H-KL designed the protocol for the study. DL and H-KL performed statistical analysis and interpretation of the data. J-DS and DL composed the article, while YW, XZ, and X-LS revised it. H-LT, FW, and Y-WW participated in the data collection and cleaning. All authors participated in significant paper revisions and approved the final manuscript.

## Funding

The current study was supported by grants from the National Natural Science Foundation of China (no 81802740 to J**-**DS; no. 81972857 to YW), Science-Health Joint Medical Scientific Research Project of Chongqing (no 2022ZDXM028 to J**-**DS), Natural Science Foundation Project of Chongqing (no cstc2018jcyjAX0741 to J**-**DS).

## Conflict of interest

The authors declare that the research was conducted in the absence of any commercial or financial relationships that could be construed as a potential conflict of interest.

## Publisher’s note

All claims expressed in this article are solely those of the authors and do not necessarily represent those of their affiliated organizations, or those of the publisher, the editors and the reviewers. Any product that may be evaluated in this article, or claim that may be made by its manufacturer, is not guaranteed or endorsed by the publisher.

## Abbreviations

NPC: nasopharyngeal carcinoma
URRBMI: Urban and Rural Residents Basic Medical Insurance
UEBMI: Urban Employee Basic Medical Insurance
EBV-DNA: plasma Epstein–Barr virus DNA load
A/G: serum albumin to globulin ratio
LAR: lactate dehydrogenase to albumin ratio
NLR: peripheral blood neutrophil count to lymphocyte count ratio
PLR: peripheral blood platelet count to lymphocyte count ratio
CI: confidence interval
OR: odds ratio
HR: hazard ratio
